# Durability Assessment and Microstructure of High-Strength Performance Bricks Produced from PET Waste and Foundry Sand

**DOI:** 10.3390/ma14195635

**Published:** 2021-09-28

**Authors:** Frank Ikechukwu Aneke, Bankole Osita Awuzie, Mohamed M. H. Mostafa, Chikezirim Okorafor

**Affiliations:** 1 College of Agriculture, Engineering and Science Howard College Campus, University of KwaZulu-Natal, Durban 4004, South Africa; 2 Department of Built Environment, Faculty of Engineering, Built Environment and Information Technology, Central University of Technology, Free State (CUT), Bloemfontein 9300, South Africa; bawuzie@cut.ac.za; 3 Sustainable Transportation Research Group (STRg), Civil Engineering, University of KwaZulu-Natal, Durban 4004, South Africa; mostafam@ukzn.ac.za; 4 Department of Construction Management and Quantity Surveying, Durban University of Technology, Steve Biko Campus, Durban 4001, South Africa; chikezirimo@dut.ac.za

**Keywords:** bricks, foundry sands, PET waste, durability, tensile strength, compressive strength

## Abstract

Fired clay brickwork in buildings is prone to cracks and deterioration upon exposure to long-time acidic contamination and water absorption, hence decreasing the bearing capacity of masonry walls. As its contribution toward resolving this challenge, this study assessed the durability and morphological characteristics of high-strength performance bricks produced from a mixture of PET waste (PW) and foundry sand (FS). The PET waste bricks (PWBs) were produced through different proportioning (PW: FS) of 20%, 30%, and 40% of the dry mass of FS. The PWBs produced were tested for durability and compressive and tensile strengths and compared to fired clay bricks to evaluate their load-bearing capacity under compression and tension. Furthermore, scanning electron microscopy (SEM) tests were employed to analyze the morphological structure of the bricks. The test results revealed that the PWBs recorded an appreciable strength of 1.5–2 times that of fired clay bricks, and lower water absorption whilst retaining their ultimate strengths after complete immersion in water and acidic concentrations. The morphology of PWB possessed greater intercluster bonds on the surface compared to clay bricks. The findings demonstrate a reasonable methodological approach toward the production of masonry bricks using a mixture of PET waste and spent foundry sands.

## 1. Introduction

Urban population growth has brought in its wake, an increase in the amount of household and municipal wastes being generated therein. Society is currently grappling with the management of these wastes, as a significant proportion of these end up in landfills. This is especially the case with plastic waste, of which polyethylene terephthalate (PET) forms a major part [1,2,3]. To buttress the debilitating impact of PET waste on the environment, these scholars maintain that it takes an estimated 400 years to naturally decompose. Scholars admit that PET waste continues to pose a challenge to developing and developed countries alike [4]. According to Rhodes [5], countries in the Sub-Saharan Africa (SSA) region have been responsible for generating an average of 180 million tons of municipal waste daily with plastic waste contributing an estimated 17 million tons. Based on the available literature, it could be concluded that various studies have been published on the effective management and challenges of municipal solid wastes in South Africa without investigating the effects of waste conversion to construction materials [6,7,8]. Aneke and Shabangu [9], in their study, concluded that municipal solid wastes, particularly scrap plastic waste and recycled crushed glass, could be used in the production of high-performance masonry bricks with very low CO_2_ embodiment. Whilst decrying the lack of effective waste management strategies and skillsets in SSA, the authors advocate material recovery and reuse through waste conversion building construction, due to its economic benefits [10,11]. South Africa is situated in SSA and is currently struggling with effective waste management [12]. Resorting to landfilling and incineration as predominant waste management methods in South Africa has been identified as having the potential to not only undermine environmental sustainability but also pose as an enabler of climate change through the emission of greenhouse gases (GHGs) into the atmosphere [13]. Although these authors acknowledge the ongoing European-styled transformations in waste management within the South African context over the past few decades, they maintain that based on the publicly available data published in 2011, just 10% of these wastes are recycled as the rest still end up either in landfills or incinerated.

Besides plastic wastes (PET), Aneke et al. [14] state that mining residues and power generation waste constitute a major proportion of waste streams prevalent in South Africa. Of significance among this category of wastes is waste foundry sand (WFS) [15]. This kind of waste results from the metal casting and molding processes, which occur in foundries and consist of a large percentage of high-quality uniformly sized silica sand was used by Aneke and Shabangu [15] to produce high performance paving bricks without compromising the environmental pollution standard. In South Africa, the WFS, which has been classified as a hazardous waste material, is disposed of in landfills after several cycles of reuse, thereby, posing a challenge to the nation’s environmental sustainability aspirations even in the face of limited landfill spaces.

Recently, the linear approach to construction material management has been criticized for making salient contributions to the unsustainable production and consumption patterns being experienced in the industry. Studies have further pointed out that construction and demolition waste (CDW) accounts for at least 30% of the total solid waste produced around the world, and the percentage is expected to increase over the next few years, because dumping these wastes in sanitary landfills has always been the traditional approach to waste management, but this will not be feasible in the years to come. To significantly reduce or eliminate the amount of CDW being dumped, the adoption of circular economy principles has been recommended as a possible solution to the increasing amounts of CDW [16,17,18,19]. The adoption of circular economy principles has been referred to as a closed-loop system or industrial symbiosis, wherein the waste product from one industry or industrial sector is deployed as a resource in another industry for production.

The construction industry’s leaning toward the adoption and implementation of circular economy practices has been heralded as a step in the right trajectory. These practices have shown potential to ameliorate the incidence of poor waste management through the introduction of efficient material handling regimes that support material recovery, recycling, and reuse for the housebuilding subsector. The extant literature is replete with reportage of cases wherein PET waste has been used: for substituting aggregates in the concrete mixture [20], improving the thermal response of unfired lightweight clay bricks with the aid of high-density polyethylene (HDPE) plastic waste [21], as aggregate replacement in blocks (plastic aggregate blocks, for the construction of (flexible) pavements [22,23], and as aggregate replacement in asphalt concrete mixture [24]. Similarly, scholars have elucidated instances wherein WFS has been utilized for the development of green-efficient masonry bricks based on a mixture of scrap plastic waste (SPW) [15], for the production of sustainable bricks, blocks, and paving stones. Alonso-Santurde et al. [25] concluded in their study that spent foundry sand can be recycled into clay bricks and there are no relevant technological drawbacks, but the feasibility strongly depends on the properties of the raw materials, as spent foundry sand may be introduced into bricks up to 30% by weight to achieve the desired strength and durability. In furtherance, waste such as crushed glass (CG) has proven to be a replacement for coarse aggregate. Thus, the replacement of coarse aggregate with CG in concrete has been reportedly sustainable, as this waste material enhances the strength of concrete paving blocks, with the replacement level ranging from 10% to 40%, for all concrete paving blocks mixed with crushed glass, as higher strength and lower water absorption were achieved within this mixed proportion with natural aggregates. At a replacement level of crushed glass of 20%, the concrete paving blocks had the highest compressive strength and the lowest water absorption. However, the tensile splitting strength of concrete blocks mixed with crushed glass reached the maximum value when the replacement level was 40%. As such, the highest performance of the block’s abrasion resistance was observed when the replacement level of CG was 30% [26,27], as a durable material for subgrade filling and pipe-embedding [28], as aggregate replacement in asphalt concrete mixtures [29], and for strengthening conventional and dry-mix concretes [30]. In another study, Petri and Timo [31] explored the utility of recycled PET blends when deployed alongside demolition wastes as a construction material. From the foregoing, the utility of PET waste and WFS in facilitating industrial symbiosis within the construction industry and beyond, in general, and in the housebuilding, sector is no longer in doubt as it has been elucidated severally in the literature. However, its usefulness within the South African context for curbing the incidence of acidic contamination and water absorption in load-bearing walls of residential dwellings remains largely underexplored. Fired clay bricks, which happen to be prevalent in the construction of residential dwellings, are prone to cracks and deterioration upon exposure to long-time acidic contamination and water absorption. These cracks and deterioration levels often culminate in a significant decrease in the bearing capacity of a building’s walls, thereby, undermining the structural integrity of such buildings. With the increasing agitation for sustainable innovative and affordable housing across various country-contexts, South Africa inclusive [32], it has become imperative to devise ways to deal with this kind of challenge. This is the gap that this study seeks to contribute toward bridging. As its central objective, this study assesses the utility of PET waste and waste foundry sand (WFS) reinforced bricks (PWBs) in tackling the incidence of acidic contamination and deterioration of load-bearing walls in residential dwellings. Furthermore, it carries out a comparison between the characteristics of conventional fired bricks and the PWB through increasing the empirical evidence of durability, strength, and microstructure as well as conversion of South African wastes (i.e., PET waste and waste foundry sand) to green-efficient high-performance brick without compromising non-replenishable natural materials as good balance is maintained between economic, environmental, and social considerations by producing masonry bricks with very low CO_2_ embodiment.

This manuscript is subdivided into three sections as such: Section 1 represents the abstracts and literature. Section 2 deals with the methods used for material collection, preparation, testing, and analysis. In Section 3, the results of the various tests conducted are presented and discussed. Section 4 details the conclusion.

## 2. Materials and Methods

**Figure 1:**
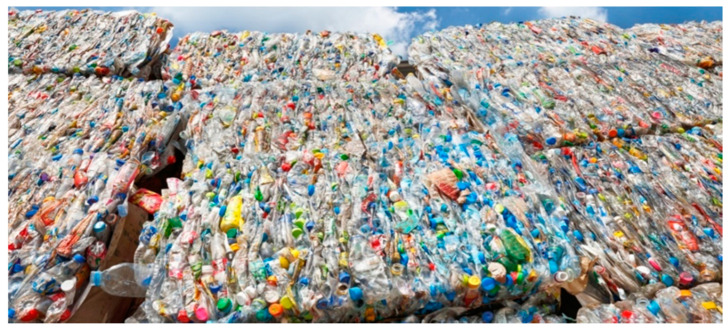
PET stockpile at Mariannhill landfill site in Durban.

Plastics are one of the growing sources of municipal solid waste (MSW) as PET plastics are found in all major MSW categories. However, PET has become an important commercial polymer with its application spanning across fabrics, molded parts for automotive, electronics, packaging, and many more. The PET waste was sampled from a landfill site in Durban, following stern COVID-19 protocols. Subsequently, the waste was conveyed to the laboratory as shown in Figure 1, followed by sanitizing and washing to eliminate any form of a virus.

**Figure 2:**
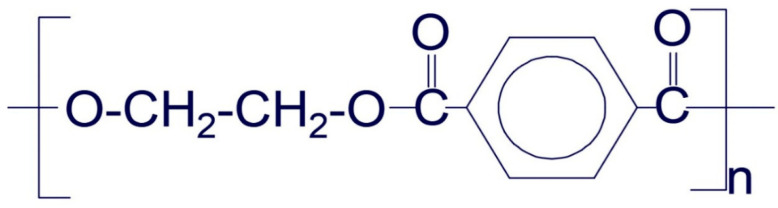
Molecular bond structure of polyethylene terephthalate waste.

PET consists of long hydrocarbons chain with carbon, hydrogen, and oxygen (C_10_H_8_O_4_)n as the dominant molecular elements. The molecular bond structure of the PET waste is presented in Figure 2. The chemical constituents of the PET waste were evaluated through an X-ray fluorescence (XRF) test. This test result revealed that the PET waste herein consisted of butylene, ethylene, and propylene with its dry density varying within 910 and 913 kg/m^3^ in conformance with ASTM D792 [33] protocol.

Foundry sand (FS) is the discarded sand from a metal casting company, this company uses river sand in the building of casting formwork. However, this river sand is considered a waste material after multiple usages. The grain size of the foundry sand used in this study was between 0.01- and 2.5-mm ASTM D1140-17 [34] sieve sizes. The FS was subjected to XRF testing to determine and evaluate the constituent chemicals available in the sand. Based on the XRF test results, the dominant compound identified at the amorphous phase of the FS was CaO, chromium (III) oxide (Cr_2_O_3_), iron (III) oxide (Fe_2_O_3_), and aluminum oxide (Al_2_O_3_) making up to 40% of the FS, whereas silicon dioxide (SiO_2)_ made up the remaining 60% of the FS according to percentage dry mass.

### 2.1. Plastic Waste Brick Preparation

**Figure 3:**
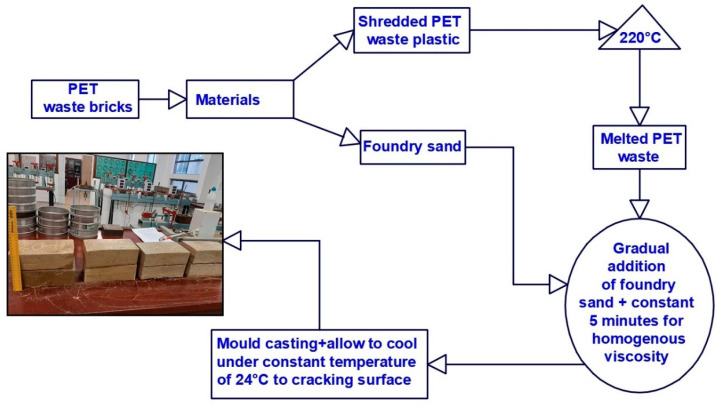
Process flow diagram of SPW bricks making.

The preparation of plastic waste bricks started with the shredding of plastic to allow for easy melting under a lesser temperature. Prior to melting of the plastic, a trial mix design of waste plastic and FS was achieved using a mix ratio of 80%:20%, 70%:30%, and 60%:40% of the dry mass of the FS and PW. The mixture of 80% foundry sand and 20% plastic waste was coded to PWB-1, which stands for plastic waste bricks. The ratios 70%:30% and 60%:40% were designated as PWB-2 and PWB-3, respectively. These mixtures were kept separately to maintain consistency throughout the study. The waste plastic was subjected to melting at a controlled temperature of 220 °C using a furnace capable of producing 800 °C of heat. Subsequently, the gradual addition of foundry sand with constant mixing commenced up to 5 min until a homogenous consistency of paste-like viscosity was obtained. Then the homogenous blend was cast into a greased mold of 222 mm length × 106 mm depth × 73 mm height to eliminate any form of adherence to the walls of the mold. Immediately after casting the blend of paste-like viscosity into the mold, compression stress of 200 kPa was applied to the casted bricks in the mold to eliminate voids as well as to densify the bricks. The produced bricks specimens were allowed to cool for 8 h before demolding commenced. As such, the bricks were allowed to continue cooling under a constant temperature of 24 °C prior to strength and durability testing. The flow process diagram for the production of the plastic waste bricks is presented in Figure 3.

The commercially available fired clay used herein was supplied by a brick producing company known as Corobrik. To maintain consistency with size, the fired clay brick obtained from the brick company had uniform dimensions compared to that of plastic waste produced for this study. The supplied clay bricks could be described as well burnt, very hard, solid in texture, and sharp in shape and color. It was also observed that the clay bricks could be easily broken under a free fall of 1.5 m.

**Figure 4:**
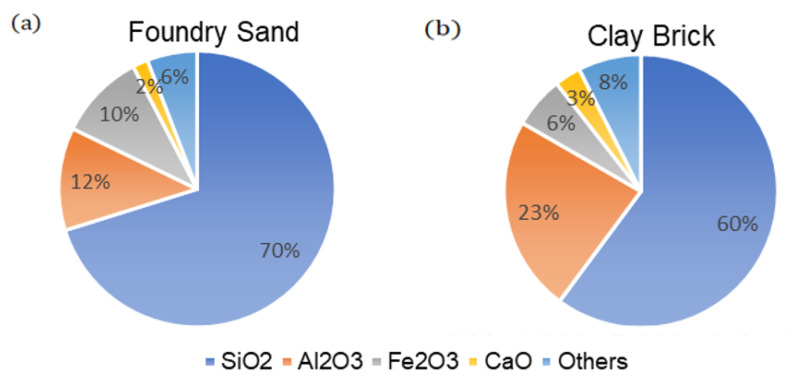
Pie chart of the chemical composition of the materials used (**a**) foundry sand, (**b**) clay brick.

The chemical constitution of commercially supplied bricks was determined using an XRF equipment machine. The procedure commenced when some fractions of the clay bricks were obtained using a hammer and rod. To maintain consistency during the XRF testing, these fractions of bricks were taken from all sides of the brick sample. After which, these fractions were pulverized using a ball milling machine following the commencement of the XRF testing. After the testing, the test results of all the materials used herein are presented in Figure 4.

### 2.2. Testing Methodology

#### 2.2.1. Unconfined Compressive Strength (UCS) Test

The unconfined compressive strength test was conducted following the ASTM C67/C67M-21 [35]. The crushing test was carried at a loading rate of 1.25 mm/min toward the depth direction using a compression machine. The upper and lower surfaces of the bricks were cleaned to remove any solid particles that influence the test result. The ultimate load at which the tested bricks failed with vertical deformation load was captured from and stored in the electronic data logger with the corresponding display of the stress–deformation curve. 

#### 2.2.2. Tensile Strength Test

The produced bricks were tested according to the ASTM C67/C67M-21 [35] protocol for the testing of bricks under tension. At the bottom surface of the tensile strength testing machine, steel of 4 mm thickness with 90 mm width and 190 mm length was placed in a horizontal orientation to ensure even distribution of pressure during crushing. The same loading rate used for the compressive strength test was applied for the tensile strength testing, and the values of tensile strength were recorded using an average of two bricks for each test with the mean value as the final value as obtained using Equation (1).

(1) $$\;\sigma_{t}=\frac{2P}{\pi .D.T}$$

The *P* cited in Equation (1) is equivalent to the maximum recorded load, while the letter *D* as cited in Equation (1) is the width of the specimen. The *T* is equated to the length of the specimen.

#### 2.2.3. Durability Test

**Figure 5:**
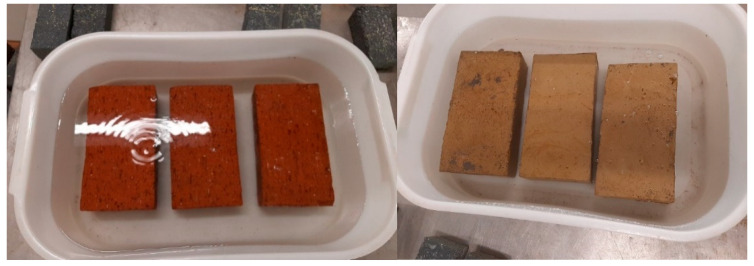
The wetting and drying cycle durability test setup for the clay and PW bricks.

The durability test commenced 3 days after the PW bricks were produced; hence, the testing was conducted in accordance with ASTM D559/D559M [36] testing procedures. To determine the resistance capacity of the PWB in an acidic environment, the bricks were completely soaked in a solution of tetraoxosulphate VI acids (H_2_SO_4_) with various concentrations of $2.3\times 10^{-5}\;M,5.2\times 10^{-4}\;M,3.6\times 10^{-3}\;M,\;\mathrm{and}\;4.6\times 10^{-2}\;M$, as shown in Figure 5. A weighing balance capable of carrying 10 kg was used to measure the dry densities of the bricks before and after 90 days. Following the soaking and weighing process, the bricks were kept dry for 24 h, and the absorption rate capacity was measure as the compressive and tensile strengths of the bricks were determined to evaluate the effects of acid soaking on the strength of the bricks.

The concentration of the H_2_SO_4_ was adjusted using water at different ratios, the water was placed in a bath while the acid was gradually poured into the bath until the concentration of the acid attenuated to the desired concentrations. This exercised was performed at a controlled temperature of 18 °C due to the exothermic reaction of the H_2_SO_4_. Subsequentially, the bricks were immersed in the acid. However, the concentration of acid solutions with their corresponding pH values is presented in Table 1.

#### 2.2.4. Initial Rate of Absorption (IRA)

The IRA is an important parameter for the durability performance of bricks in a damp/moist environment. This test is usually performed following the ASTM C67/C67M-21 [35] testing procedures. The IRA parameter of bricks is measured by first driving out moisture from the brick voids through oven drying followed by recording the dry mass of the brick specimens. A graduated cylinder was used to measure out 10 mL of water, which was then poured into a pan followed by the partial submersion of the brick base to a height of 7 mm. The submerged bricks were left in the water bath for one minute; subsequently, the water was retrieved and placed back into the graduated cylinder. As such, the IRA of the brick was calculated through the amount absorbed after 1 min of submersion.

#### 2.2.5. Scanning Electron Microscopy (SEM)

The SEM test is an important procedure devoted to analyzing the morphology of the composite matrix. The VEGA3 TESCAN-6480 machine was operated at a voltage of 20 kV following the ASTM E986-04 [37] testing protocols. A representative sample of each brick was placed in the SEM machine, as the microscope’s detection of 1 µm was used for the identification of unknown particles as well as for revealing the microstructure of the brick samples. The SEM test also provides the basis for the analysis of interactions between substances and their corresponding substrates.

## 3. Results

### 3.1. Unconfined Compressive Strength of Bricks

**Figure 6:**
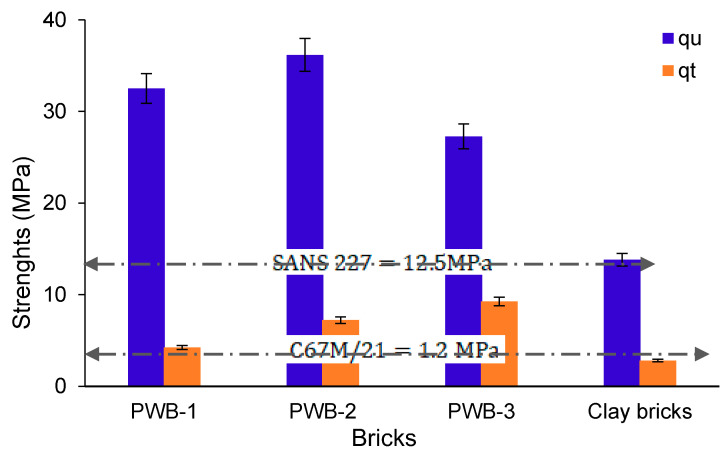
The q_u_ and q_t_ of studied bricks.

The UCS of the plastic waste bricks was tested against compression and tension, 3 days after production alongside the strengths of fired clay bricks, as presented in Figure 6. The strength of plastic waste bricks under compression was twice greater than that of commercially supplied fired clay bricks. It is worthy to mention that the strength of a single brick unit could significantly influence the brick prism for the design of masonry structure as well as increase the flexural elastic modulus of the structure.

Remarkably, it is noted that the strength of the produced plastic waste bricks recorded compressive strength values of 33.12, 36.18.01, and 28.40 MPa for PWB-1, -2, and -3, respectively, as compared to the fired clay bricks that recorded 13.81 MPa on average. Based on the compressive strength results, the strength rendered by PW bricks complies with the South African standard (SANS 227:2007) [38] for clay bricks. The standard stipulates that a minimum compressive strength of 17.0 MPa is required for face prism bricks, whereas 12.5 MPa worth of strength is stipulated for a brick unit. However, the commercially available bricks and PWBs comply with SANS. The standard stipulated strength is valid for the load-bearing capacity of storied buildings with other retaining load-bearing structures. In furtherance, the densities of 1784, 1887, 1828, and 1894 kg/m^3^ were obtained for PWB-1, -2, -3, and clay bricks, respectively. The developed high strength rendered by PWB is mobilized by the morphology of the viscoelastic properties as the plastic waste melts under high temperature (220 °C), thereby, forming a much stronger matrix composite with foundry sand. It is also evident that the bricks produced with 70% foundry sand and 30% PET waste recorded the highest strength; beyond this mix ratio, the strength dropped. This implies that the percentage of foundry sand significantly influenced the strength of the PWBs. Other than the foundry sand’s influence on the plastic bricks, the utilized precompression stress also mobilized the strength development by reducing porosity through composite densification [39]. It was also noted that the tension resistance of PWB-3, which contains 40% of PET plastic, is higher compared to that of the rest of the bricks. The high tensile value rendered by this brick is mobilized by the ductile and viscoelasticity of the melted PET plastic. Generally, the PWBs recorded an average of 57% increase under compression as juxtaposed with the compressive strength of fired clay-fired bricks used herein. The strength result produced in the study herein agrees with the investigation reported by Gumaste et al., [40], which stated that incorporation of PET in the production of composite bricks increased the compressive and tensile strength of the composite and as such contributed to a significant decrease of 40% CO_2_ emissions without using sand and cement.

### 3.2. Effect of Acid Soaking on the q_u_ and q_t_ of the Bricks

**Figure 7:**
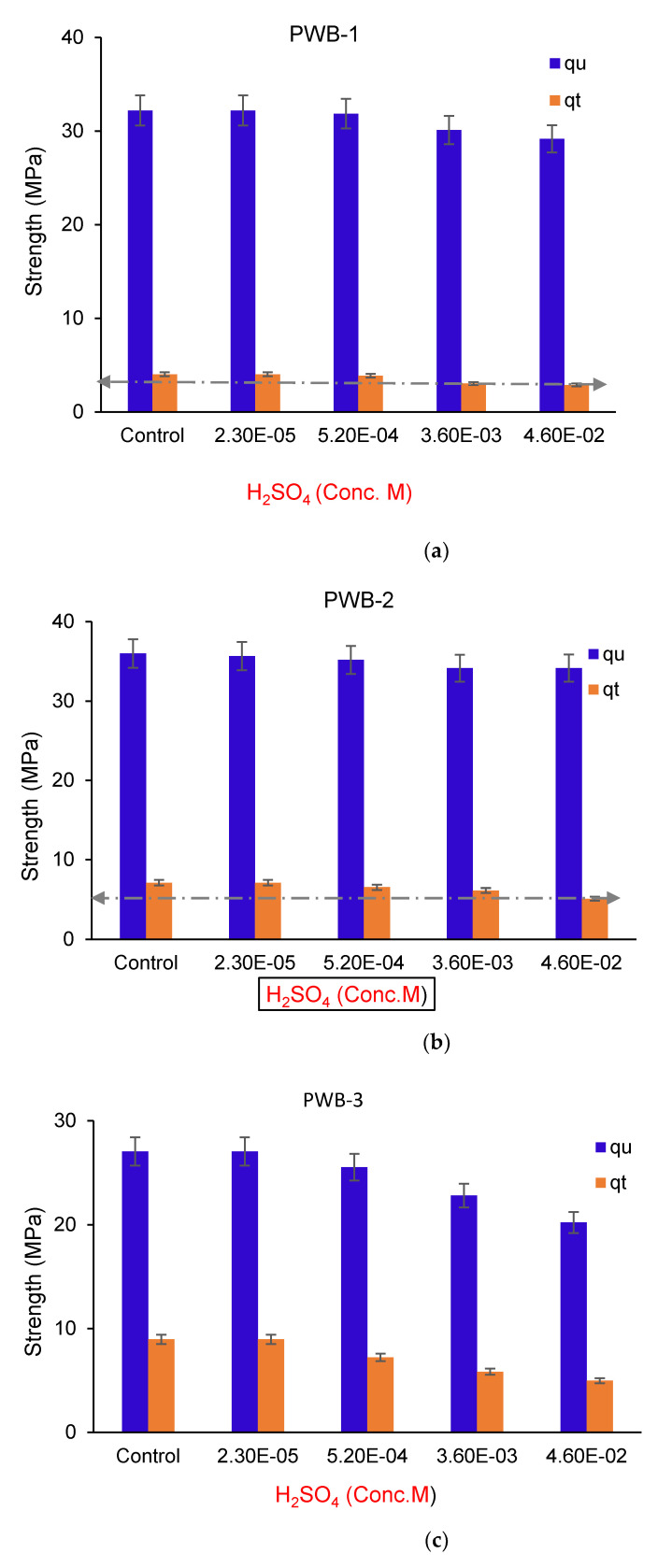

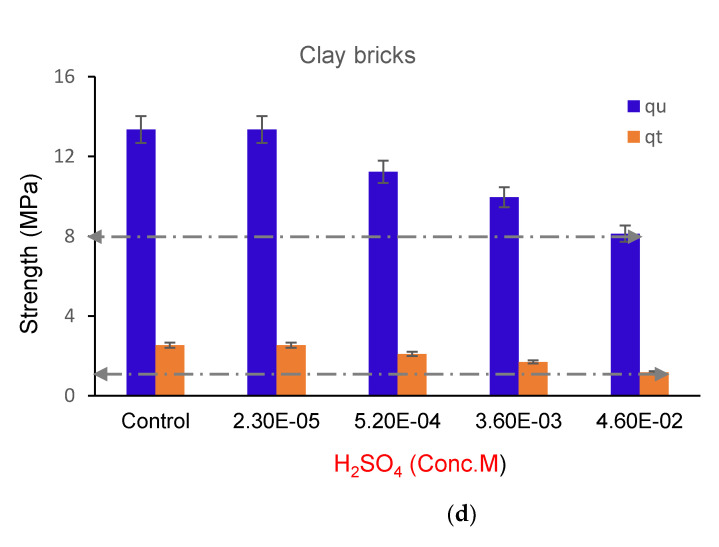
(**a**) Effects of acid concentrations on tension and compression resistance of PWB-1. (**b**) Effects of acid concentrations on tension and compression resistance of PWB-2. (**c**) Effects of acid concentrations on tension and compression resistance of PWB-3. (**d**) Effects of acid concentrations on tension and compression resistance of clay brick.

Most of the clay bricks used for construction in an acidic environment deteriorate within a short time frame, due to the interaction between clay minerals and the acidic environment. Hence, the reaction will trigger the impairment of this structure, causing it to not reach its service life. Under this context, the performance of the plastic waste bricks produced in this study was compared with that of commercially obtained fired clay bricks using acid soaking as the environmental index. Based on tested results as presented in Figure 7a–d, the acidic soaking caused less than a 2% decrease in strength for PWB-3, whereas the percentage decrease in strength for PWB-2 and -1 were 4.25% and 6.12%, respectively. However, the decrease in strength has a great proportionality between the percentages of the plastic waste used to produce each set of bricks. It is noted that the bricks with a higher percentage of plastic waste portrayed higher resistance to acidic soaking, while the bricks with lesser contents of plastic showcased lesser resistance to acid attack. At the highest acid concentration, $4.6\times 10^{-2}\;M$, PWB-3 portrayed more hydrophobic performance due to its higher percentage content available in the bricks. Generally, it was noted that the acidic soaking had slight effects on the plastic bricks due to a higher hydrophobic tendency, unlike in the fired clay bricks, which recorded over 15.12% decrease in strength due to 90 days of acidic soaking interactions. This decrease in strength was mobilized by acid adsorption within the void spaces of the clay bricks triggering cation exchange replacement through proton coupling with the diffusion within pores of the bricks. The interaction between the fired clay bricks and H_2_SO_4_ acidic solution caused the decrease of the brick’s alkalinity, therefore, leading to loss of stability and hydrolysis through the destruction of the internal microstructure of the fired clay bricks leading to a significant reduction in strength as reported elsewhere [38,40].

The standard deviation of the plastic waste bricks was calculated in this study using the compressive and tensile strength values of the produced bricks to determine how closely the strength values were related, using an average of two bricks for each strength test with the mean value as the final value. Equation (2) was used for the formulation and determination of standard deviation value for the produced high-performance bricks.

(2) $$S=\sqrt{\frac{\sum^{\;}{\left(x-\bar{x}\right)}^{2}}{\eta -1}}\;$$

where S is the standard deviation; x is equivalent to compressive and tensile strength values, and η is the number of tested bricks.

The standard deviation result of the tested brick strength was obtained to be 13.32. The obtained standard deviation is sufficiently far away from zero, and this implies that the strength of the tested bricks was more spread out. Furthermore, the test pointed out that the means obtained present a statistically significant difference between waste plastic bricks and fired clay bricks. These analyses show that the compressive strength increases with increase of PET waste up to 30% inclusion of PET, beyond which, the strength decreased. Table 2 presents the summary of the mean and standard deviation values of the bricks.

### 3.3. Interdependence of the q_u_ and q_t_ of the Bricks

**Figure 8:**
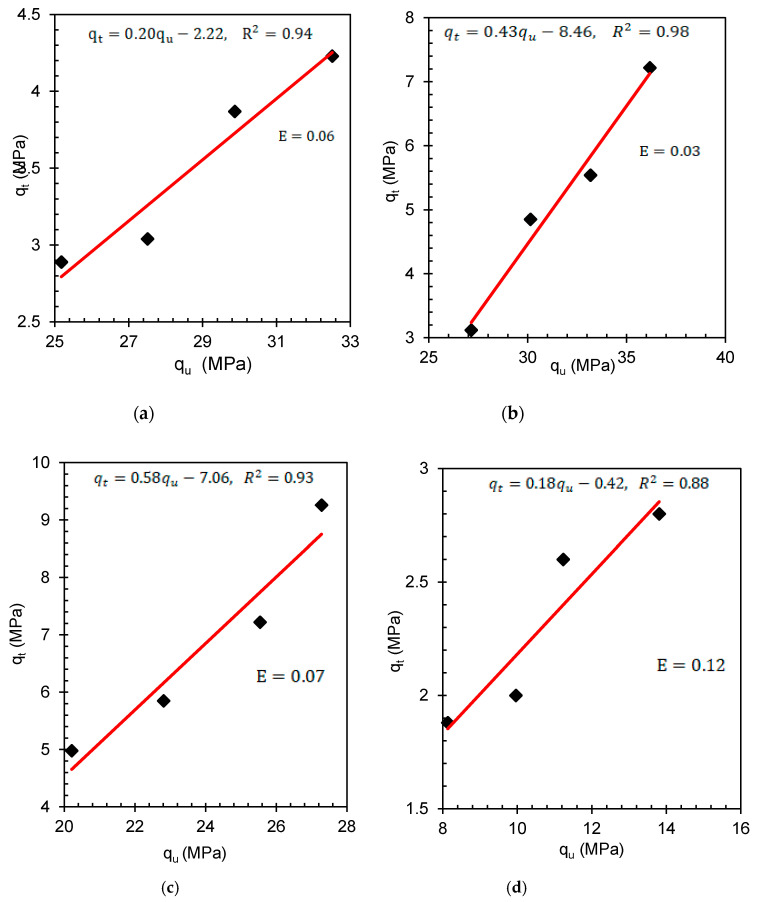
(**a**–**d**) Interdependence of q_t_ and q_u_ of PWB-1, PWB-2, PWB-3 and clay bricks brick composites for strength indices.

The basic structural design elements depend on the correlation between the compressive and tensile strength values of the consistent material. The masonry-retaining structures could be subjected to flexural stress. Under these circumstances, the combined effects of compression and tension could trigger shear failure; therefore, reliance on the interdependence of compression and tension strength of the bricks produced here is required [41,42]. Therefore, the interdependence of compression and tension strength of the studied bricks is presented in Figure 8a–d. The curves indicate a bi-linear relationship between tensile and compressive strengths with a corresponding coefficient of determination (R^2^) of 0.94, 0.98, 0.93, and 0.88 for PWB-1, PWB-2, PWB-3, and clay bricks, respectively. The high R^2^ values are mobilized by the ratios of plastic waste and foundry with regard to apparent porosity, which is dependent on strength. According to Wight and MacGregor [41] and McCormac and Brown [42] the ratio of q_t_ and q_u_ of M20 grade concrete should be 10%; hence, the ratio between q_t_ and q_u_ for the produced bricks was evaluated to be 12.17%, this implies that the produced plastic bricks could sustain flexural stress.

### 3.4. Load–Deformation Relationship and Failure Modes

**Figure 9:**
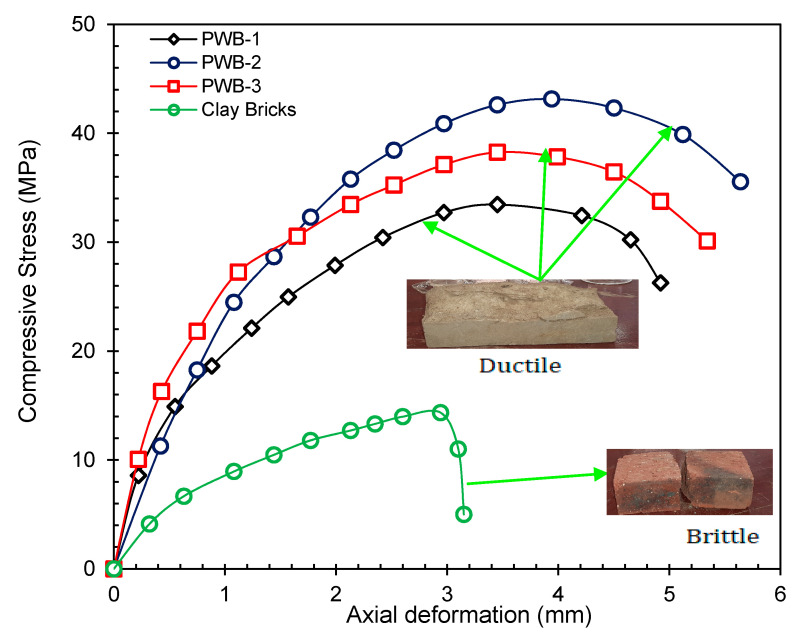
Load–deformation relationship of PW and fired clay bricks.

The compression stress–strain response of the plastic waste and fired clay bricks is shown in Figure 9. It is noted that the plastic waste bricks portrayed ductile failure type of stiffness under compression compared to their counterpart the fired clay bricks that exhibited significant brittle behavior. However, it was observed that the ductile behavior was mobilized by the viscoelasticity properties of the melted plastic waste, whereas the brittle response of the clay brick was due to a great proportionality between the clay minerals and sintering temperature of heat used during the clay bricks production. At a strain value of 4.12 mm, the PWB rendered significant ductile stiffness, with corresponding stress relaxation between strain gauge values of 1.1 to 2.05 mm. The brittle failure response of the clay bricks under compression was observed as a sudden drop after peak load. The stress–strain behavior of all the bricks studied here is mobilized by the linear correlation between the compression load and deformation to the strain gauge value of 3.6 mm; beyond this value strain, hardening was observed only in the plastic waste brick while free fall was recorded on the fired clay bricks as they were completely crushed at a strain gauge value of 2.91 mm without residual strain resistance. The plastic waste bricks portrayed multiple planes of failure intention prior to its final failure. As such, this confirmed that the plastic waste bricks were reliably ductile, whereas the clay bricks investigated in the study are brittle matrices. The presented test result in this study agrees with the investigation published elsewhere by Gumaste *et al.* [40], Knutson [43]; and Ewing and Kowalsky [44]. The multiple planes of failure observed in plastic waste bricks could be attributed to percentages of the melted plastic waste due to the viscoelasticity of the bricks that triggered elongated deformation.

### 3.5. Initial Rate of Absorption (IRA)

**Figure 10:**
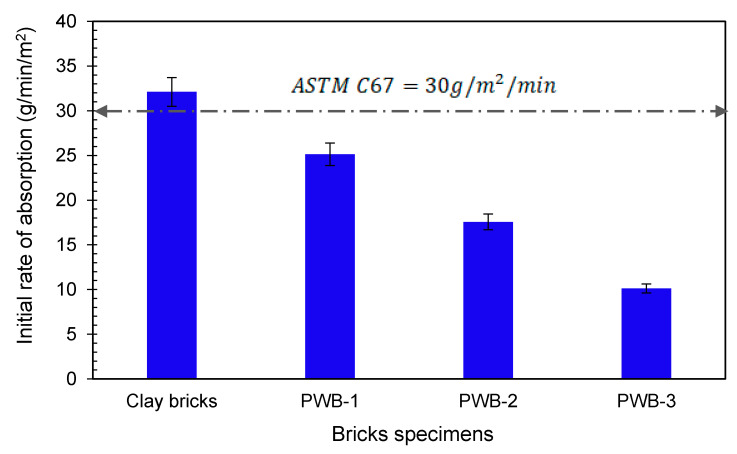
Initial rate of absorption for the produced brick specimens.

The IRA of both the plastic waste and fired clay bricks is presented in Figure 10. The IRA test result for the fired clay bricks in this study was evaluated to be 32 g/m^2^/min. Similarly, for PWB-1, -2, and -3 the rendered IRA values were 25.14, 17.57, and 10 g/m^2^/min, respectively. However, for a brick to have good adhesion strength, the IRA values must exceed 30 g/m^2^/min according to Yorkdale [45]; therefore, a study was adopted using ASTM C67 [35]. In furtherance, an IRA value greater than 30 g/m^2^/min implies that the brick unit is highly absorptive and should be wetted prior to laying to achieve adequate bond strength, though the IRA limit values were derived based on tests carried out on clay bricks. Additionally, Knutson [43] reported that the IRA values for fly ash brick varied from 35 to 50 g/m^2^/min with an average of 40 g/m^2^/min. Based on the obtained test result, it could be concluded that bricks produced using plastic waste and foundry sand in this study could completely be used as an alternative to clay brick, without influencing the fundamental properties of the bricks as stipulated by the ASTM C67 [35]. However, it could be understood that the value of IRA is found to be higher in fired clay bricks compared to plastic waste bricks. Regardless of the bricks’ IRA values, an adequate bond between bricks and mortar could be achieved with plastic waste bricks produced in this study since initial absorption through wetting before laying is not required because their IRA values are within the allowable limit. Thus, the IRA gives an insight into the pre-wetting time needed and the bond strength of brick masonry. It was noted that the initial rate of absorption for brick specimens incorporating plastic waste and foundry sand rendered lower IRA values compared to that of fired clay bricks; as such, the produced fired clay bricks in this study require pre-wetting for an adequate brick–mortar bond.

### 3.6. Scanning Electron Microscopy (SEM)

**Figure 11:**
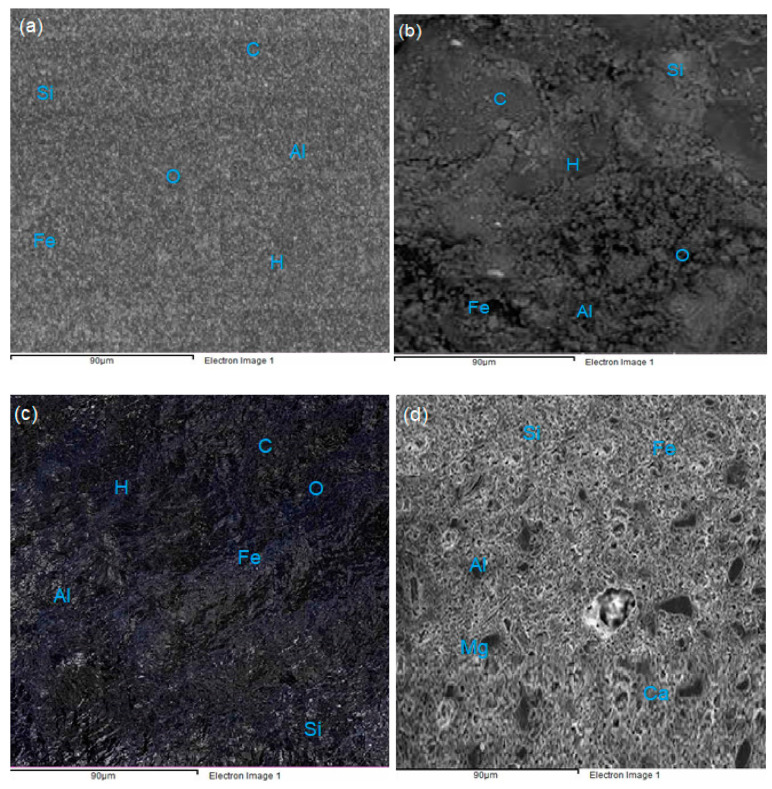
(**a**–**d**) The micrographs obtained by SEM of PW and fired clay bricks.

The morphological comparative study was conducted on PW and fired clay brick specimens, using an SEM apparatus VEGA3 TESCAN-6480 scanning electron microscope operated at 20 kV. The morphology of PW bricks with different percentages of PET waste as well as the microstructure of the fired clay bricks is presented in Figure 10. The morphologies of the PWB-1, -2, and -3 are given in Figure 11a–c. It is noted that the bricks with 20% of melted PET are grayish, the gray color gradually changed to black as the PET content increased, as can be seen in the figures. The PW bricks portrayed viscous floccules with foundry sand forming a tight matrix structure sealed within the surface and the inner part of the bricks. The morphology showed traces of quartz as identified by the white spots on micrographs, whereas the black and grayish patches with a glassy and unwrinkled surface were identified as ethylene and propylene that serve as binders. The same chemical compounds were identified on PWB-2 and PWB-3 with more of a blackish shinning microstructure. This was an indication of higher percentages of melted PET plastic used in the production of the bricks. Significant, changes were observed on the PWB because of the PET waste inclusion that coated and knitted the sand particles, as well as filling the pore spaces within the matrix structure. The chemical composition identified on the surfaces of the PW bricks was as follows: carbon C, hydrogen H, and oxygen O, which are rendered by PET plastics, while Si, Fe, and Al were the dominant elemental compound from foundry sand. The inclusion of melted plastics in the PW bricks resulted in the reduction of pore space within the bricks, and this observation is particularly pronounced in PWB-2 and -3. The pore spaces in the bricks were minimized drastically causing them to relatively possess greater strength.

In Figure 11c, the EDx of the fired clay bricks suggested the presents of aluminum, silicon, magnesium, and calcium as the primary elements. The designated dark areas are the partially burnt clay particles shown by irregular black porous parts. Whereas the grayish identified areas are the mixture of calcium and silicon, which appeared to be spherical with a small bulging of siliceous and aluminous glass. Generally, the pore spaces in fired clay bricks were greater compared to those in the PW bricks that rendered more of a knitted solid matrix structure within the bricks.

### 3.7. Sustainability Analysis of PET Waste Bricks

The sustainability analysis was achieved through a CO_2_ emission comparison between the fired clay and plastic waste bricks to quantify the CO_2_ embodiment of both the plastic and clay bricks when used for the construction of two rooms. The mix formulation used in the production of the bricks is presented in Section 2.1. However, it is presumed that cost of extraction, sorting out and transportation of clay, plastic waste, and foundry sand will be the same; therefore, these costs were not considered in this analysis. The cost of producing energy invested in the process of producing these bricks for the construction of a two-bedroom masonry structure was used for this analysis based on the CO_2_ embodiment. Further, the same quantity of mortar will be required for the masonry construction for both the plastic and clay bricks; therefore, the energy cost of mortar was excluded in this analysis.

According to Eskom [46], the cost of electricity for households and businesses, which includes all components of the electricity bill such as the cost of power distribution and taxes is ZAR 2.38 and ZAR 1.83 per kWh. This is equivalent to USD 0.145 and USD 0.070 per kWh. Therefore, 1 kWh of electricity is equivalent to 1895.63 °C of heat. Based on the calculation analysis performed in this study, 0.58 kWh will be required to generate 1100 °C worth of heat to produce 1 fired clay brick unit, whereas 0.12 kWh will be required to generate 220 °C worth of heat to produce 1 unit of plastic waste brick. Based on the energy production cost for fired clay bricks, the cost for 1 brick unit is ZAR 1.38 (ZAR 2.38 × 0.58 kWh), while the plastic waste brick will cost ZAR 0.286 (ZAR 2.38 × 0.12 kWh). To evaluate the quantities of CO_2_ embodiment for both the production of fired clay and plastic waste bricks. In South Africa, coal-based power plants emit an average of 915 g of carbon dioxide (CO_2_) per kilowatt-hour of electricity produced. It has been calculated that 0.58 and 0.12 kWh of electricity are required to produce 1 unit of both fired clay and plastic bricks, respectively. Therefore, to calculate the quantities of CO_2_ emission for clay brick, (915 g × 0.58 kWh) 531 g of CO_2_ will be emitted into the environment, whereas (915 g × 0.12 kWh) 110 g of CO_2_ will be discharged into the environment to produce plastic bricks. According to the calculated bill of material quantity presented in this study, 23,534 pieces of bricks will be required for the construction of a two-room masonry structure of 20 m in length, 3 m width, and 4 m height, using double leaf wall with the exclusion of two doors and two windows spaces. Each brick weighs an average of 3.3 kg; therefore, (23,543 × 3.3) 77,662.2 kg of clay would be required to produce 23,534 pieces of clay bricks. Therefore, the total quantities of CO_2_ in kilograms that will be discharged into the environment for the construction of a two-bedroom masonry structure are (0.531 kg × 77,662.2 kg) 41,239 kg worth of CO_2_. To calculate the quantity of CO_2_ embodiment for the production of plastic bricks in kilogram, as known, 30% of plastic waste inclusion rendered the ultimate strength, therefore, the quantities of material required for the construction of a two-bedroom masonry structure will be 30% multiplied by the quantities of material required for the construction multiplied by the quantity of CO_2_ emission required for a single unit of plastic waste brick (0.3 × 77,662.2 kg × 0.11 kg), and this corresponds to 2563 kg of CO_2_ emission. The summary of the estimation of the bricks’ quantity of CO_2_ emission is presented in Table 2. It is worth mentioning that the calculated CO_2_ emission for both fired clay and plastic waste bricks is based on the kilograms of energy required and CO_2_ embodiment cost to produce these bricks. Using the information provided in Table 3, it follows that over (0.3 × 77,662.2 kg) 23,299 kg of plastic waste could be diverted from landfills, compared to about 77,662.2 kg of clay required for the construction of a two-room structure, with a consequent saving of 41,239 kg of carbon dioxide.

## 4. Conclusions

In this research, the development of plastic waste brick was studied through the indices of strength, durability, microstructure, as well CO_2_ embodiment. Based on the findings of this study, the inclusion of scrap plastic in the production of masonry bricks using blends of PET waste and foundry sand could be considered an effective way and a cost-effective approach toward the conversion of dwindling natural clay to produce sustainable and green-efficient masonry bricks.

The utilization of PET waste and foundry as raw materials for the manufacturing of masonry bricks is one of the rational ways of recycling abundant wastes, leading to the conservation of space, water, and soil. The PET waste and foundry sand used in this study were abundantly available waste materials, having a high contribution to environmental pollution. Resistance against acidic attack is commendable for the PWB-1, -2, and -3, indicating their suitability in a moist environment due to their high resistance capacity because of their hydrophobic properties.

The utilization of plastic waste with varying percentages of foundry sand is proven in this study as a resourceful option for load-bearing brick masonry. The bricks also portrayed great potentiality against acidic attacks. It was observed that the blend of 30% PW and 70% foundry sand of plastic bricks resulted in 36.18 MPa with approximately 60.31% increase in strength compared to fired clay bricks. These compressive strength values of plastic bricks satisfy the requirement of compressive strength specified by South African standard for burnt clay masonry units (SANS 227, 2007), which requires nominal compressive strength for face bricks to be greater than 17.0 MPa, with individual strengths greater than 12.5 MPa, for burnt clay brick with a load-bearing capacity of retaining walls and storied buildings.

Furthermore, it was observed that the initial rate of absorption of the fired clay bricks was higher compared to that of PWB-1, -2, and -3. It can be concluded that the plastic waste content of plastic waste bricks triggered a decrease in water absorption and IRA. The fired clay bricks showed the highest water absorption among all the produced bricks in this study due to the high content of clay minerals and double-layer structure, which absorbs more water to fill up the structure. The SEM revealed that the microstructure of PWB-1, -2, and -3 were mobilized with lower porosity; therefore, the plastic waste bricks require no form of pre-wetting before their use during masonry construction.

## Figures and Tables

**Table 1:** Acidic submersion of bricks.

Series	Concentration (M)	H_3_O^+^	pH	Soaking Time (Days)
1	2.30 × 10^−5^	2.30 × 10^−5^	4.64	90
2	5.20 × 10^−4^	5.20 × 10^−4^	3.28	90
3	3.60 × 10^−3^	3.60 × 10^−3^	2.44	90
4	4.60 × 10^−2^	4.60 × 10^−2^	1.34	90

**Table 2:** Standard deviation of the bricks.

Bricks	Compressive Strength (MPa)		Tensile Strength (MPa)		$\mathrm{Mean}\;\bar{x}$	$S=\sqrt{\frac{\sum^{\;}{\left(x-\bar{x}\right)}^{2}}{\eta -1}}$
-	1st trial	2nd trial	1st trial	2nd trial	15.56	13.32
PWB-1	32.58	31.84	4.03	3.28		
PWB-2	37.00	35.00	7.88	6.32		
PWB-3	25.22	28.88	8.32	9.60		
CB	6.00	8.00	2.00	3.08		

CB: clay bricks.

**Table 3:** Sustainable and embedded CO_2_ analysis of bricks.

Bricks	Heat (°C)	Production Time	Average Density (kg/m^3^)	CO_2_ Emission (kg)	Price/Energy Cost	Sustainability
PWB-1	220	(Minutes)	1887	1373	ZAR 0.286/brick	Favorable
PWB-2	220	10	1828	2060	ZAR 0.286/brick	Favorable
PWB-3	220	10	1784	2746	ZAR 0.286/brick	Favorable
Clay bricks	1100	10	1894	41,239	ZAR 1.34/brick	Not Favorable

## Data Availability

Data is contained within this article and in a private repository. It can be made available upon request.

## References

[1] Aslani H., Pashmtab P., Shaghaghi A., Mohammadpoorasl A., Taghipour H., Zarei M. (2021). Tendencies towards bottled drinking water consumption: Challenges ahead of polyethylene terephthalate (PET) waste management. Health Promot. Perspect..

[2] Ayeleru O.O., Dlova S., Akinribide O.J., Ntuli F., Kupolati W.K., Marina P.F., Blencowe A., Olubambi P.A. (2020). Challenges of plastic waste generation and management in sub-Saharan Africa: A review. Waste Manag..

[3] Aryan Y., Yadav P., Samadder S.R. (2019). Life Cycle Assessment of the existing and proposed plastic waste management options in India: A case study. J. Clean. Prod..

[4] Perera S., Arulrajah A., Wong Y.C., Horpibulsuk S., Maghool F. (2019). Utilizing recycled PET blends with demolition wastes as construction materials. Constr. Build. Mater..

[5] Rhodes C.J. (2018). Plastic pollution and potential solutions. Sci Prog..

[6] Dlamini S., Simatele M.D., Serge Kubanza N. (2019). Municipal solid waste management in South Africa: From waste to energy recovery through waste-to-energy technologies in Johannesburg. Local Environ..

[7] Simatele D.M., Dlamini S., Kubanza N.S. (2017). From informality to formality: Perspectives on the challenges of integrating solid waste management into the urban development and planning policy in Johannesburg, South Africa. Habitat Int..

[8] Guerrero L.A., Maas G., Hogland W. (2013). Solid waste management challenges for cities in developing countries. Waste Manag..

[9] Aneke F.I., Celumusa S. (2021). Strength and durability performance of masonry bricks produced with crushed glass and melted PET plastics. Case Stud. Constr. Mater..

[10] Barra R., Leonard S.A., Whaley C., Bierbaum R. Plastics and the Circular Economy. https://www.thegef.org/sites/default/files/publications/PLASTICS%20for%20posting.pdf.

[11] Aneke F.I., Awuzie B. (2018). Conversion of industrial wastes into marginal construction materials. Acta Structilia.

[12] Serge Kubanza N., Simatele M.D. (2020). Sustainable solid waste management in developing countries: A study of institutional strengthening for solid waste management in Johannesburg, South Africa. J. Environ. Plan. Manag..

[13] Godfrey L., Oelofse S. (2017). Historical review of waste management and recycling in South Africa. Resources.

[14] Aneke F.I., Okonta F.N., Ntuli F. (2015). Geotechnical Properties of Marginal Highway Backfill Stabilized With Activated Fly Ash. Master’s Dissertation.

[15] Aneke F.I., Shabangu C. (2021). Green-efficient masonry bricks produced from scrap plastic waste and foundry sand. Case Stud. Constr. Mater..

[16] Ginga C.P., Ongpeng J.M.C., Daly M., Klarissa M. (2020). Circular economy on construction and demolition waste: A literature review on material recovery and production. Materials.

[17] Lederer J., Gassner A., Kleemann F., Fellner J. (2020). Potentials for a circular economy of mineral construction materials and demolition waste in urban areas: A case study from Vienna. Resour. Conserv. Recycl..

[18] Tomaszewska J. (2020). Polish transition towards circular economy: Materials management and implications for the construction sector. Materials.

[19] Mahpour A. (2018). Prioritizing barriers to adopt circular economy in construction and demolition waste management. Resour. Conserv. Recycl..

[20] Ismail Z.Z., Al-Hashmi E.A. (2008). Use of waste plastic in concrete mixture as aggregate replacement. Waste Manag..

[21] Limami H., Manssouri I., Cherkaoui K., Saadaoui M., Khaldoun A. (2020). Thermal performance of unfired lightweight clay bricks with HDPE & PET waste plastics additives. J. Build. Eng..

[22] Vasudevan R., Sekar A.R.C., Sundarakannan B., Velkennedy R. (2012). A technique to dispose waste plastics in an ecofriendly way–Application in construction of flexible pavements. Constr. Build. Mater..

[23] Gürü M., Çubuk M.K., Arslan D., Farzanian S.A., Bilici I. (2014). An approach to the usage of polyethylene terephthalate (PET) waste as roadway pavement material. J. Hazard. Mater..

[24] Hassani A., Ganjidoust H., Maghanaki A.A. (2005). Use of plastic waste (poly-ethylene terephthalate) in asphalt concrete mixture as aggregate replacement. Waste Manag. Res..

[25] Alonso-Santurde R., Andrés A., Viguri J., Raimondo M., Guarini G., Zanelli C., Dondi M. (2011). Technological behaviour and recycling potential of spent foundry sands in clay bricks. J. Environ. Manag..

[26] Patil A.R., Sathe S.B. (2020). Feasibility of sustainable construction materials for concrete paving blocks: A review on waste foundry sand and other materials. Mater. Today Proc..

[27] Yeo J.S., Koting S., Onn C.C., Mo K.H. (2021). An overview on the properties of eco-friendly concrete paving blocks incorporating selected waste materials as aggregate. Environ. Sci. Pollut. Res..

[28] Arulrajah A., Yaghoubi E., Imteaz M., Horpibulsuk S. (2017). Recycled waste foundry sand as a sustainable subgrade fill and pipe-bedding construction material: Engineering and environmental evaluation. Sustain. Cities Soc..

[29] Bakis R., Koyuncu H., Demirbas A. (2006). An investigation of waste foundry sand in asphalt concrete mixtures. Waste Manag. Res..

[30] de Matos P.R., Marcon M.F., Schankoski R.A., Prudêncio L.R. (2019). Novel applications of waste foundry sand in conventional and dry-mix concretes. J. Environ. Manag..

[31] Petri S., Timo K. (2019). Recycled construction and demolition waste as a possible source of materials for composite manufacturing. J. Build. Eng..

[32] Moghayedi A., Awuzie B., Omotayo T., Le Jeune K., Massyn M., Ekpo C.O., Braune M., Byron P. (2021). A Critical Success Factor Framework for Implementing Sustainable Innovative and Affordable Housing: A Systematic Review and Bibliometric Analysis. Buildings.

[33] ASTM D792-20 (2020). Standard Test Methods for Density and Specific Gravity (Relative Density) of Plastics by Displacement.

[34] ASTM D1140-17 (2017). Standard Test Methods for Determining the Amount of Material Finer than 75-μm (No. 200) Sieve in Soils by Washing.

[35] ASTM C67/C67M-21 (2021). Standard Test Methods for Sampling and Testing Brick and Structural Clay Tile.

[36] ASTM D559/D559M-15 (2015). Standard Test Methods for Wetting and Drying Compacted Soil-Cement Mixtures.

[37] ASTM E986-04(2017) (2017). Standard Practice for Scanning Electron Microscope Beam Size Characterization.

[38] SANS 227:2007 and SANS 1 575 (2007). The South African National Standard for Burnt Clay Paving Units.

[39] ASTM C67 (2003). Standard Test Methods for Sampling and Testing Brick and Structural Clay Tile.

[40] Gumaste K.S., Nanjunda R.K.S., Venkatarama R.B.V., Jagadish K.S. (2007). Strength and elasticity of brick masonry prisms and wallets under compression. Mater. Struct..

[41] Wight J.K., MacGregor J.G. (2012). Reinforced Concrete: Mechanics and Design.

[42] McCormac J.C., Brown R.H. (2015). Design of Reinforced Concrete.

[43] Knutson H.H. (2003). The stress-strain relationship for masonry. Mason. Int..

[44] Ewing B.D., Kowalsky M.J. (2004). Compressive behaviour of unconfined and confined clay brick masonry. J. Struct. Eng..

[45] Yorkdale A., Borchelt J. (1982). Initial Rate of Absorption and Mortar Bond. Masonry: Materials, Properties, and Performance.

[46] (2009). ESKOM 2000–2008-Our Recent Past-“Shift performance and Grow Sustainably”. www.eskom.co.za.

